# What X‐Ray Absorption Spectroscopy Can Tell Us About the Active State of Earth‐Abundant Electrocatalysts for the Oxygen Evolution Reaction**

**DOI:** 10.1002/anie.202211949

**Published:** 2022-11-15

**Authors:** Marcel Risch, Dulce M. Morales, Javier Villalobos, Denis Antipin

**Affiliations:** ^1^ Nachwuchsgruppe Gestaltung des Sauerstoffentwicklungsmechanismus Helmholtz-Zentrum Berlin für Materialien und Energie GmbH Hahn-Meitner-Platz 1 14109 Berlin Germany; ^2^ Institut für Materialphysik Georg-August-Universität Göttingen Friedrich-Hund-Platz 1 37077 Göttingen Germany

**Keywords:** Electrocatalysis, Manganese Oxides, Operando Spectroscopy, Oxygen Evolution Reaction, X-Ray Absorption Spectroscopy

## Abstract

Implementation of chemical energy storage for a sustainable energy supply requires the rational improvement of electrocatalyst materials, for which their nature under reaction conditions needs to be revealed. For a better understanding of earth‐abundant metal oxides as electrocatalysts for the oxygen evolution reaction (OER), the combination of electrochemical (EC) methods and X‐ray absorption spectroscopy (XAS) is very insightful, yet still holds untapped potential. Herein, we concisely introduce EC and XAS, providing the necessary framework to discuss changes that electrocatalytic materials undergo during preparation and storage, during immersion in an electrolyte, as well as during application of potentials, showing Mn oxides as examples. We conclude with a summary of how EC and XAS are currently combined to elucidate active states, as well as an outlook on opportunities to understand the mechanisms of electrocatalysis using combined operando EC–XAS experiments.

## Graphical abstract

Electrochemical methods and X‐ray absorption spectroscopy are powerful tools to understand electrocatalysis on an atomistic level, yet the combination of these methods still holds untapped potential.

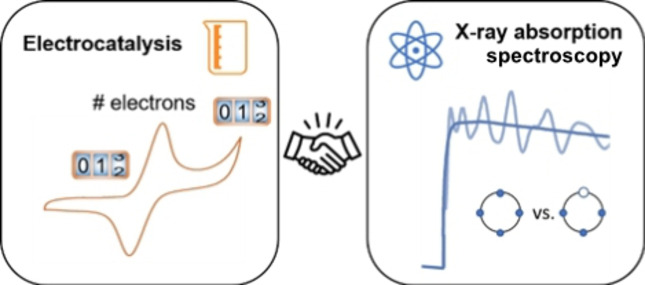

## 1 Introduction

A sustainable energy supply is of global interest and one of the grand societal challenges of the century.[^1^, ^2^] The UN defined it as one of its 17 goals for sustainable development, namely, to “ensure access to affordable, reliable, sustainable and modern energy for all”.^[3]^ One crucial target is to increase the use of renewable energy, which necessitates storage to buffer the intermittence of renewable sources such as wind and sun. Chemical energy storage is an attractive solution. It requires an electrocatalyst that provides reaction intermediates to lower the energy barriers, thereby increasing the storage efficiency. In order to rationally improve electrocatalyst materials and thus the reaction efficiency, the nature of the electrocatalyst under reaction conditions must be revealed.[^4^, ^5^] Unfortunately, many important details of the active states of electrocatalysts under reaction conditions are still unknown.[^6^, ^7^]

For a better understanding of electrocatalysts, the combination of electrochemical (EC) methods and X‐ray absorption spectroscopy (XAS) has been very insightful. In contrast to spectroscopic and diffraction methods, XAS is element‐specific, which allows correlating EC processes to oxidation state changes of specific elements and which further allows elucidating the changes in the coordination environment of these elements. For the latter, no long‐range order is required when using XAS as compared to XRD. This renders XAS suitable for the study of electrocatalysts, which lose their crystallinity during the formation of the active state.[^8^, ^9^, ^10^, ^11^] Akin to XRD, XAS can be made more surface sensitive by measuring at grazing incidence.[^12^, ^13^] Zhu et al.^[14]^ recently reviewed the features and limitations of in situ XAS and in situ XRD and the readers are directed there for further comparison. Another advantage of XAS for electrochemical in situ experiments, e.g., over IR spectroscopy,^[15]^ is the comparably low interference of the incoming and outgoing photons with water. Thus, the advantages of XAS over other methods enable much untapped potential to understand EC processes in combined EC and XAS experiments. Several aspects of combined EC and XAS experiments have been reviewed in the last decade approached from the perspective of the synchrotron science community.[^14^, ^16^, ^17^, ^18^, ^19^, ^20^] Here, we will complement the prior work by approaching the topic and its synergetic aspects from the perspective of an electrochemist.

What is measured in electrochemical experiments? In these experiments, electric current and voltages (defined as the difference between electrode potentials) are measured, where the former is the flow of electrons and the latter quantifies the capacity of the electrochemical system to do work. Common pitfalls in the physical interpretation of typical electrode potentials used in electrochemistry were recently discussed by Boettcher et al.^[21]^ The application of an electrode potential may lead to various effects on an electrocatalyst material. For instance, it could induce a flow of electrons with only little change to it, e.g., metallic conduction via an electron gas. It may induce a chemical change in the material, such as a change in oxidation state of the metals or, in some cases, of the ligand oxygen. The latter changes may lead to complete transformation of the electrocatalyst, often as corrosion. The potential‐induced changes may take place on the surface (defined here as the liquid–solid interface, including inner pores), near the surface or in the bulk.

What is measured by XAS? In these experiments, a core electron is excited leaving a so‐called core hole in an inner shell, which makes the method highly sensitive to the local geometric and electronic structure through various secondary processes. The X‐ray absorption near‐edge structure (XANES) correlates with the number of electrons in the outermost shell (i.e., the oxidation state) via screening effects of the created core hole. Structural information is more straightforwardly extracted from the extended X‐ray absorption fine structure (EXAFS), which arises due to interference of the ejected core electron with itself. A higher energy of the X‐ray photon results in a larger penetration depth in the material and a larger escape depth being relevant for proxies of the X‐ray absorption measured, e.g., by the X‐ray fluorescence. For the materials and absorption edges covered herein, these depths (calculation in Supporting Information) are of the order of 0.3 μm (soft XAS, typically <1 keV) to 60 μm (tender/hard XAS, typically >5 keV) for simple Mn oxides. Since nanosized materials are attractive electrocatalysts due to their high surface to volume ratio, the XAS discussed herein probes, with some exceptions discussed later, the entire electrocatalyst material.

**Figure 1:**
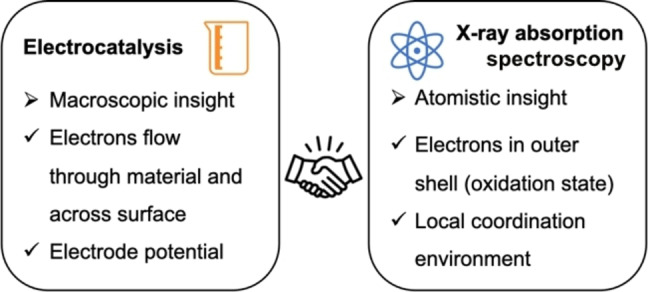
Key insight from X‐ray absorption spectroscopy and electrochemistry.

Figure 1 compares the insights that can be gained from combined EC and XAS experiments, which offer several synergies. Electrochemistry provides macroscopic insights into the behavior of the electrochemical cell and its components, where the working electrode is host to the catalyst under investigation and thus the most interesting. This is complemented by an atomistic insight into the electrocatalyst by XAS. The most valuable synergy is given by the ability to follow electrons through a combination of EC and XAS, where EC measures the flow rate of electrons, i.e., a current, while XAS is sensitive to the oxidation state, that is, the number of electrons in the outer shells, which determines the reactivity of the elements in the electrocatalyst. This is particularly insightful when coupled with additional electrochemical or non‐electrochemical detection of the product of interest so that it becomes possible to correlate the current due to product generation with an oxidation state change, thus removing ambiguities about the origin of the electrons used for the formation of the desired product.

In this Minireview, we concisely introduce the basics of EC and XAS, providing the necessary framework to discuss changes of the electrocatalyst material. As a way of example, we focus on manganese oxides as electrocatalysts for the oxygen evolution reaction (OER), which is the anodic reaction in water electrolyzers and currently considered the efficiency‐limiting process in these devices.^[4]^ We emphasize the importance of considering that changes may occur not only during a catalytic reaction, but also during preparation, storage, immersion in an electrolyte, or when applying potentials even if those do not lead to catalytic reactions. We conclude with a concise summary of how EC and XAS are currently combined to elucidate the active state of an electrocatalyst as well as an outlook on future opportunities for an in‐depth understanding of the mechanisms of electrocatalysis.

## 2 Brief Fundamentals of Electrochemistry

Electrochemistry relates chemical reactions to moving charges in response to gradients in the electric or chemical potential.^[22]^ Any discontinuity between two materials may produce opposing charges at the interface; the (electrical) double layer, where charge transfer reactions take place. Within the scope of this Minireview, we will only discuss the double layer in the liquid near a solid–liquid interface. When the potential difference is larger than the free energy of a given reaction, charge transfer becomes possible and ions, either in the liquid near the electrode surface or in the solid, may change their oxidation state by a redox reaction. In order for charge transfer to occur, the electrons need to be given sufficient energy to overcome kinetic and thermodynamic barriers between different states of the electrocatalysts, e.g., the barrier for Mn oxidation. A simple model for the relation between the difference in electrode potential and reaction rate (or current density) of a simple redox reaction is given by the Butler–Volmer equation.^[23]^ Yet, catalytic reactions typically require multiple charge transfers of different types, e.g., the OER needs the transfer of four electrons and protons (acid) or hydroxide ions (base). The half reactions of the OER are:

(1) $$\mathrm{Acid}\;\;2\;H{}_{2}O\to O{}_{2}+4\;e{}^{-}+4\;H{}^{+}$$

(2) $$\mathrm{Base}\;\;4\;\mathrm{OH}{}^{-}\to O{}_{2}+4\;e{}^{-}+2\;H{}_{2}O$$

Several proposals exist for possible intermediates.^[24]^ Often the assumption is made that the reaction rate of one of the intermediates is significantly lower than those of the other steps, which defines the rate‐limiting step (RLS) and reduces the complex multi‐charge transfer reaction to a simpler reaction with a single charge transfer.^[25]^ The state prior to the slow step is also the most likely to be resolved by XAS (and other complementary methods).

Electrochemical experiments, in particular those during combined EC and XAS experiments, are either performed by holding the potential at the working electrode for a certain time and measuring the current (chronoamperometry) or by holding the current at the working electrode and measuring the voltage (chronopotentiometry). In addition, cyclic voltammetry (CV) is a convenient potentiodynamic electrochemical method to probe and possibly distinguish processes with charge transfer (Faradaic processes) and those without charge transfer (non‐Faradaic processes).^[26]^ In this method, the electrode potential is swept back and forth within a potential window and the current is measured. By convention, sweeping towards more positive potentials means sweeping in the anodic direction and sweeping toward more negative potentials corresponds to the cathodic direction. Thereby, the method combines a thermodynamic property, namely potential, with kinetics measured as an electric current. Note that for the study of half reactions such as the OER, one often uses a third electrode to sense the potential near the electrode of interest (working electrode) and lets the current flow between the latter and an auxiliary electrode (also called counter electrode). The advantage of this three‐electrode setup is that the measured current can be attributed fully to the reaction at the working electrode.

**Figure 2:**
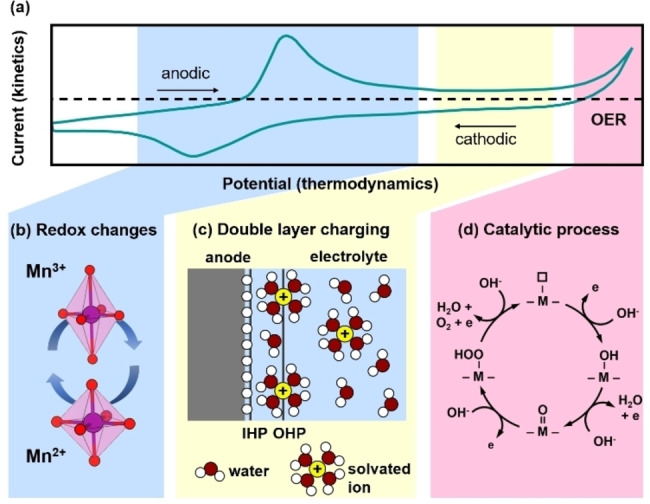
Anatomy of a cyclic voltammetry trace and associated physical processes. a) Cyclic voltammogram of electrodeposited MnO_*x*_ and assignment of the electrochemical processes of b) transition metal redox changes that may change the coordination environment, c) double layer charging, and d) electrocatalysis, here OER. Panel (a) was modified from ref. [27] with permission from the ACS. Panel (c) was modified from ref. [23] with permission from Wiley.and Sons. Panel (d) was reproduced from ref. [28] under a CC BY NC ND 4.0 License.

Figure 2 shows a typical CV experiment on an electrodeposited manganese oxide related to birnessite.^[27]^ In this example, the potential region highlighted in blue in Figure 2a shows the fingerprint of a (quasi) reversible redox reaction on an oxide in a liquid, in this case, the redox transition from Mn^2+^ to Mn^3+^ in the anodic direction, and the corresponding reduction of Mn^3+^ to Mn^2+^ in the cathodic direction (Figure 2b, Table 1). The potential in the middle of the peaks is called the midpoint potential. Possible structural changes are schematically shown for the first coordination shell, where a Jahn–Teller distortion could occur due to oxidation of Mn^2+^ (d^5^ high spin) to localized Mn^3+^ (d^4^ high spin), thus leading to a change in apical bond lengths. Jahn–Teller distortions have been well studied, e.g., for LiMn_2_O_4_,^[29]^ where it is well observed at temperatures below room temperature but not above.[^29^, ^30^, ^31^, ^32^]

**Table 1:** Typical analyses of the discussed potential regions.

Potential region	Typically analyzed for
Redox changes	Redox pairs Number of active sites Phase changes
Double layer	Surface area Capacitance
Electrocatalysis	Electrocatalyst activity, stability, selectivity Catalytic mechanism

The potential region indicated with a yellow background in Figure 2a shows a capacitive behavior, i.e., an increase in potential results in an unchanging current due to constant charge accumulation in the double layer that can be modeled as a plate capacitor (Figure 2c). Therefore, the charge increases with area and the data obtained in this region can be used to determine the area of the material in contact with the electrolyte so that the electrochemical surface area (ECSA) can be estimated (Table 1) when no Faradaic processes occur.^[33]^ It is a common method but we^[34]^ and others[^33^, ^35^, ^36^, ^37^, ^38^] have pointed out common pitfalls in its determination by voltammetric methods.

In the high potential region indicated in pink background in Figure 2a, there is an exponential increase in current in the anodic direction which, however, does not display a corresponding cathodic peak. This is a typical fingerprint of an irreversible reaction, here the OER, under the applied conditions. Several processes may contribute to the measured currents in addition to catalysis, namely the aforementioned Faradaic and non‐Faradaic processes. After collection of the catalytic currents, either or both suitably normalized currents (*i*) and their corresponding electrode potentials (*E*) are used for determination of the electrocatalyst's activity and stability for benchmarking or for determination of mechanistic parameters such as the Tafel slope, reaction order and Nernst slope (Table 1).[^37^, ^39^, ^40^, ^41^, ^42^, ^43^, ^44^] Recommendations and protocols for electrocatalyst benchmarking, including, for example, suitable correction and/or appropriate conditioning procedures, have been published by several groups;[^37^, ^39^, ^40^, ^44^, ^45^, ^46^] a harmonization of the benchmarking endeavors is desirable but has not been agreed upon yet.

For mechanistic insight, the Tafel slope (∂log*i*/∂*E*), reaction order with respect to pH (∂pH/∂log*i*) and Nernst slope (∂*E*/∂pH) are determined and compared to predictions from the proposed reaction paths.[^28^, ^47^, ^48^, ^49^] Unfortunately, the electrochemical parameters are often ambiguous, especially when the conclusions rely on a single mechanistic parameter such as the Tafel slope, since they may be influenced by concomitant processes, e.g., redox transformation of an electrocatalyst component or competing Faradaic reactions, as well as by additional factors including electrical conductivity, blocking of the electrode surface due to gas bubble formation, local pH changes, among others.[^50^, ^51^] Therefore, complementary spectroscopic investigations as well as reaction product analysis are needed to corroborate the mechanistic insights gained by electrochemical methods.

The assignment of physicochemical processes to the observed features in the CV is often unclear. In addition to redox reactions, adsorption/desorption processes on metallic surfaces can also result in peaks in the CV, where one of the best‐known examples is hydrogen underdeposition on Pt.^[39]^ The peak potential of adsorption/desorption processes depends on the concentration of the involved ion in solution, which can be used for their identification.^[22]^ On metal oxides, adsorption/desorption without a redox process is rarely discussed, likely due to localized electronic states.[^54^, ^55^, ^56^, ^57^, ^58^] Furthermore, different surface facets may have different reaction energies of the same element and thus two anodic peaks do not necessarily indicate redox of two different elements.[^59^, ^60^, ^61^] Likewise, one broad peak or shoulder does not necessarily correspond to a single redox reaction but instead may correspond to several redox reactions.^[62]^ This is exemplified in the cathodic peak in Figure 2a, which, in addition to reduction of Mn, comprises contributions from the oxygen reduction reaction (ORR, being the reverse reaction of the OER) as seen by the asymmetry of the anodic and cathodic peaks and the drop of the current below the zero‐current baseline (dashed line) at potentials more cathodic than the redox peak. The assignment of a specific redox couple is particularly challenging for multi‐metallic oxides and an additional characterization such as XAS should be used to reduce the ambiguity.

**Figure 3:**
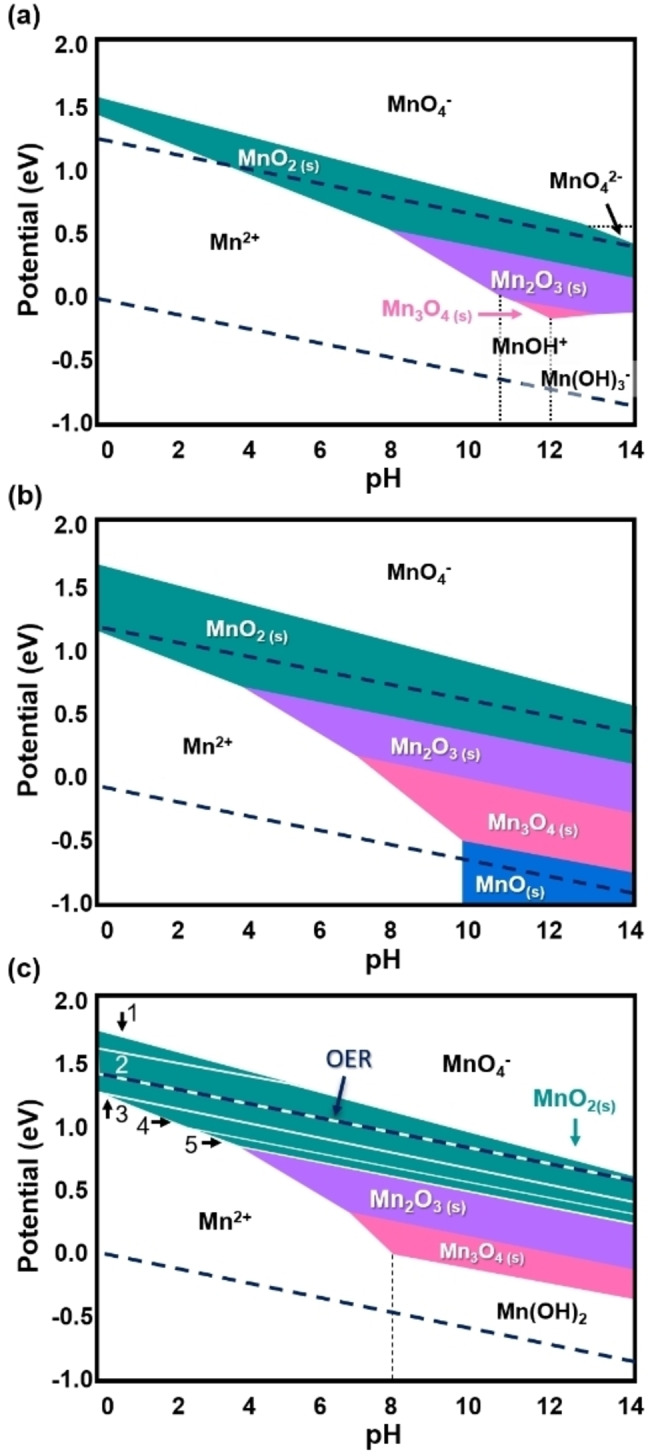
*E*–pH (Pourbaix) diagrams. a) Bulk diagram with low Mn concentration (10^−8^ mol kg^−1^ Mn). b) Bulk diagram with high Mn concentration (1 mol kg^−1^ Mn). c) Surface diagram of rutile MnO_2_ with high Mn concentration (1 mol kg^−1^ Mn) indicating surface phases 1=4O, 2=3O, 3=2O_b_, 4=O_b_+OH_b_, 5=2OH_b_. Additional information in ref. [52]. Dashed lines indicate the stability window of water between OER (upper line) and hydrogen evolution (lower line). Panels (a) and (b) were produced using materialsproject.org^[53]^ under CC BY 4.0 license. Panel (c) was modified from ref. [52] with permission from the Royal Society of Chemistry.

A redox transition may also be coupled to a phase transition in an oxide. The expected changes in the thermodynamically stable phases are displayed in *E*–pH diagrams, also called Pourbaix diagrams. Commonly, the bulk phases are calculated (Figure 3a,b) but for some cases, including the Mn−O−H system, surface phases have also been calculated, e.g., on rutile β‐MnO_2_ (Figure 3c). The calculations suggest O‐terminated MnO_2_ as the active surface phase at OER conditions (as observed by the overlap of the OER line and the MnO_2_ phase dominance in Figure 3c). While these plots are helpful to estimate what phases may be observed in an experiment, there are several important aspects that need to be considered but are not included in them. Firstly, all possible reactions are assumed to be in chemical equilibrium and possible kinetic effects are neglected. Secondly, most experimental systems are chemically more complex, e.g., the study of a binary manganese oxide in KOH corresponds to the system Mn‐K−O−H, which may stabilize additional phases such as birnessites (e.g., δ‐K_*x*_MnO_2−*y*_⋅z H_2_O) as compared to the system Mn−O−H. The cation concentration (e.g., that of Mn) determines the stability of the phases where low concentrations favor solvated species at otherwise identical pH and *E* (Figure 3a) and large concentrations favor solids (Figure 3b). Another example is the case of tunnel‐structured manganese oxides, whose properties including the framework stability may be strongly influenced by the charge‐balancing cations found within their tunnels.[^63^, ^64^] For instance, when immersed in Na_2_SO_4_ aqueous solution, the structure of cryptomelane‐type manganese oxide (a‐MnO_2_) collapses due to Na^+^ intercalation.^[65]^ Furthermore, complexation such as Mn with PO_4_^3−^ ions in a phosphate buffer is rarely explicitly included in *E*–pH diagrams, but can have a large effect on the stabilization and destabilization of phases. Calculated *E*–pH diagrams provide valuable guidance in the interpretation of the active state but additional measurements are required to fully understand possible phase changes occurring during electrochemical experiments.

## 3 Brief Fundamentals of X‐ray Absorption Spectroscopy

XAS is an element‐specific method with high chemical sensitivity that allows distinguishing different valence states of the probed elements and, in some cases, it can provide a deep insight into crystal field splitting and electronic structure. The absorption of an X‐ray photon at a specific atom, e.g., Mn, occurs when the energy of the interacting photon is equal to or higher than the binding energy of a specific core electron of the atom, leaving a core hole that triggers secondary processes. In a typical XAS experiment, the incident photon energy is stepped or swept using a monochromator. The absorption coefficient can either be obtained from the change in transmission through the sample, which is directly recorded (e.g., using ion chambers), using the Beer–Lambert law, or from the fluorescence yield or electron yield of the sample, which is recorded with suitable detectors in a geometry where these yields are proportional to the absorption coefficient.[^12^, ^66^, ^67^]

The quantized nature of the absorption process results in a sharp discontinuity in intensity as a function of energy for transitions to continuum states, which is called the edge. Edges are labeled using the X‐ray notation^[68]^ (or IUPAC notation), where capital letters K, L, M and higher are assigned to core holes produced in shells with principal quantum numbers *n*=1, 2, 3 and higher, respectively. The spectroscopic notation is also used,^[69]^ which combines the principal quantum number with a small letter, indicating the azimuthal quantum number, where s, p, d, etc. correspond to *l*=0, 1, 2, etc., respectively. For example, the Mn‐K edge indicates a core hole in a Mn‐1s shell, whereas the Mn‐L edges indicate a core hole in a Mn‐2s or Mn‐2p shell. A larger quantum number leads to shorter attenuation lengths in the sample of at least few tens of nanometers (Figure S1).^[70]^ XAS is most suitable for the analysis of samples around an attenuation length in transmission mode and thinner than an attenuation length in fluorescence mode or electron yield mode where the latter is not discussed here due to the short escape depth in water. Since the attenuation length of the other modes in the electrocatalyst material is large as compared to the ionic radii of the top layer (<0.1 nm for Mn),^[71]^ the study of surface processes requires high‐surface area samples so that a large fraction of the measured volume is located near the surface. Nanoparticles are an example with high surface to volume ratio, which are also attractive electrocatalytic materials.

**Figure 4:**
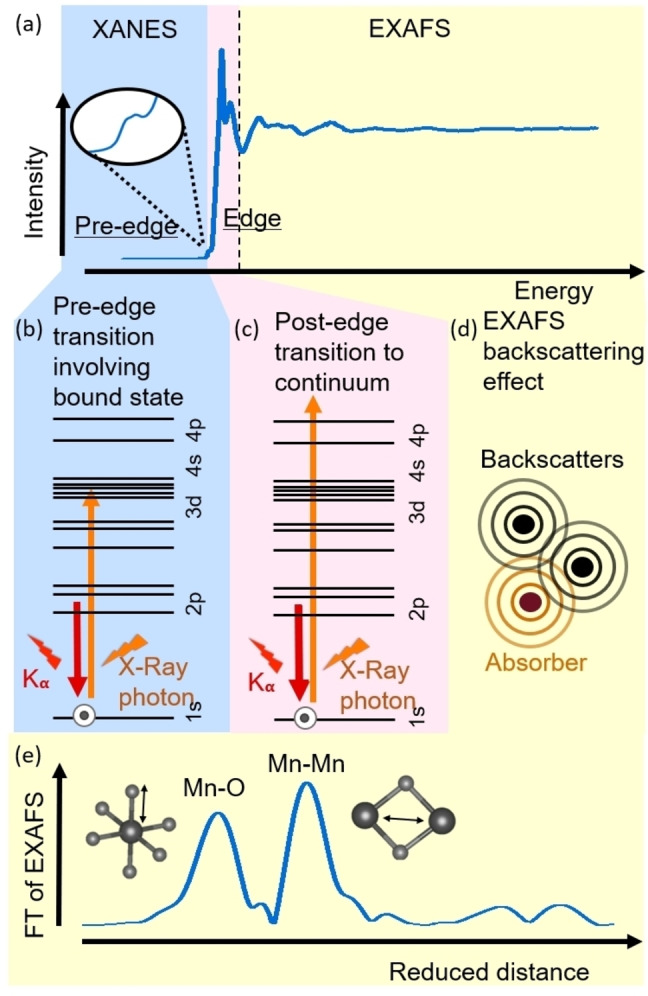
Anatomy of an X‐ray absorption spectrum and associated physical processes. a) X‐ray absorption spectrum of LiMn_2_O_4_ consisting of the XANES (blue and pink highlights) and EXAFS regions (yellow highlight). The inset magnifies the pre‐edge. Assignment of features in the spectrum to b) a pre‐edge transition to a bound state, c) a main edge transition to the continuum and d) backscattering and self‐interference that produce the EXAFS, which is often displayed as e) a Fourier transform between 15 and 600 eV where peaks can be assigned to distances between atoms. The data was recorded at beamline KMC‐3 of the synchrotron BESSY II. Dataset in ref. [72].

Figure 4a shows the X‐ray absorption spectrum of a manganese oxide, here LiMn_2_O_2_, at the Mn‐K edge, which can be partitioned into three regions: (i) the pre‐edge, (ii) the main edge, and (iii) the extended X‐ray absorption fine structure (EXAFS). The spectrum can be described by interatomic transitions to partially occupied bound states (pre‐edge; Figure 4b) or to unoccupied continuum states (main edge; Figure 4c) as well as an electron being ejected from the absorbing atom to the continuum where it scatters between neighboring atoms and interferes with itself (EXAFS; Figure 4d).^[73]^ The pre‐edge and edge regions constitute the X‐ray absorption near‐edge structure (XANES), also called near‐edge X‐ray absorption fine structure (NEXAFS).

The pre‐edges in transition metal K edges such as the Mn‐K edge are assigned to a transition from 1s states to partially occupied 3d states (Figure 4b), which is forbidden by the dipole selection rule (Δ*l*=±1). Yet, it can be observed in experimental spectra, often due to mixing of p and d orbitals^[74]^ and in rare cases due to a quadrupole transition (where Δ*l*=±2).^[75]^ For most transition metal oxides, the pre‐edges have low intensity. Notable exceptions are transition metal oxides with low symmetry in the first coordination shell and having low occupation of the d orbitals such as tetrahedral KMnO_4_ (d^0^),^[74]^ so that the pre‐edge can be used to estimate the coordination symmetry (Table 2). More often, the oxidation state is determined using the pre‐edge area or position relative to reference materials (Table 2); the accuracy of the analysis is not discussed in literature. Furthermore, contributions of *e_g_* and *t*_2*g*_ states to the pre‐edge can be quantified in some cases[^76^, ^77^] with the caveat that the transition cannot be described accurately as a single electron transition due to charge transfer and multiplet effects.^[78]^ Furthermore, the ligand K edges are usually more sensitive to *e_g_* and *t*_2*g*_ states as compared to the metal K edges.

**Table 2:** Typical analyses of the discussed spectral regions.

Spectral region	Typically analyzed for
Pre‐edge	Oxidation state Coordination symmetry
Main edge	Oxidation state
XANES	Fingerprinting/speciation Oxidation state
EXAFS	Interatomic distances Number of interactions (coordination number) (Dis)order, i.e., Debye–Waller factor

The main edge is caused by a dipole transition from 1s to unoccupied 4p continuum states (Figure 4c). A sharp maximum is often observed at the top of the edge, also known as the white line (because it showed up as a white line on photographic plates used in the early days of synchrotron research). The edge position shifts to higher energies with higher oxidation state of the absorbing metal but it also depends on the nature of the metal's ligand and the local structure.^[79]^ Two related explanations are discussed:^[73]^ firstly, oxidation (donating electrons) lowers the shielding of the core electrons which increases their effective charge so that the energy difference of the transition increases; and secondly, absorber and neighboring scattering ligands with distance *R* can be treated as a potential well, in which the energy increases as 1/*R* ^2^ (known as Natoli's rule).^[80]^ Usually, a higher formal oxidation state leads to a shorter metal–ligand distance. The empirical determination of the oxidation state by calibration of a series of well‐known reference materials is well established and has clear requirements. Firstly, the energy of the edge must be accurately estimated. Typical definitions of the edge position include the energy at 0.5 intensity in a normalized spectrum, the inflection point, and the area under the edge rise.[^67^, ^81^] Alternatively, linear combination analysis (LCA) or principle component analysis (PCA) are used,[^82^, ^83^, ^84^] which make use of the fingerprint character of the XANES. They can be more accurate as they are less dependent on, e.g., noise at the edge position but depend strongly on the parameters of the data normalization (typical normalization procedures, e.g., in refs. [50, 85]). Except for PCA, the analysis is only valid if the observed oxidation states are a combination of the reference states and no new electronic states emerge. It should be noted that typical beamline optics result in a fractional broadening (due to the energy resolution δ*E*/*E*) of about 1×10^−4^, while the broadening due to the finite core‐hole lifetime is between 2×10^−4^ to 6×10^−4^ (refs. [84, 86]) so that measurements at different beamlines have comparable broadening determined by the physics of the X‐ray absorption process. The accuracy of the calibration plot is rarely discussed, yet we have shown calibration of the Mn oxidation state of manganese oxides at the Mn‐K edge can have an accuracy of about ±0.16 oxidation states^[51]^ (rms error) using the area under the edge. It is expected that the accuracy is reduced when the core‐hole broadening becomes very large, e.g., for 2^nd^ row transition metals. The accuracy of the oxidation state calibration deserves a detailed discussion to be published elsewhere.

The oscillations at energies higher than the main edge are called EXAFS. They are caused by intensity modulations of the absorption coefficient due to electrons that make transitions to the continuum, scatter at neighboring atoms and then interfere with themselves (Figure 4d), forming constructive and deconstructive interference (observed in the spectrum as oscillations). Thus, the EXAFS encodes information about the surroundings of the absorbing atom, including the distance between the absorbing and the scattering atoms, the number of scattering atoms at a given distance and the EXAFS Debye–Waller factor, which relates to the local order (Table 2). More precisely, the most frequent (and not the average) atomic distances are measured, which is an important distinction, e.g., for Jahn–Teller distortions (e.g., of Mn^3+^), i.e., an octahedron with groups of four and two similar distances (Figure 2b). As the EXAFS is not sensitive to bond angles, often the structural insight is discussed in terms of coordination shells. Usually, the EXAFS is isolated from the edge and the energy axis is converted to momentum (wavenumber) space. Subsequently, a Fourier transform is applied which converts the momentum space axis to a real space axis, thus providing the advantage that the coordination shells appear as peaks in the typically used plots of the modulus of the complex EXAFS function (Figure 4e). These plots show the reduced distance on the *x*‐axis, which is smaller than the interatomic distances due to a contribution of the scattering phase in the Fourier transform. The interatomic distances, occupation of a given shell (i.e., coordination number) and Debye–Waller factor can be obtained by fits to the simulated EXAFS. The latter requires scattering factors and phase information that is nowadays most commonly obtained from first principles calculations performed on a suitable atomic model. Penner‐Hahn^[73]^ estimates that the interatomic distances from optimized fits can be as accurate as ±0.01 Å (<1 %) for shorter distances, e.g. metal–oxygen, while the shell occupation is only accurate to about ±1 due to the strong correlation with the Debye–Waller factor. Li, Bridge and Booth^[87]^ report errors in the amplitude (and thus coordination number) of 5–10 %. Ravel and Kelly^[88]^ point out common pitfalls in the analysis of the coordination number by EXAFS that affect accuracy. We conclude that EXAFS can determine the interatomic distances more accurately than variables derived from the amplitude such as the coordination number.

In our Fourier transform example (Figure 4e), two peaks are prominently visible, where the one at lower distance has been assigned to the Mn−O shell and the one at higher distance has been assigned to a Mn−Mn shell. Often, these assignments can be guessed with some experience based on the reduced distance but it is prudent to confirm the assignment using simulations. The EXAFS of single scattering events is usually observed to about 5 Å of the absorbing atom,[^67^, ^73^, ^85^] however, positive interference of multiple scattering events can extend it to about 8 Å (Figure S2) for metal oxides with long‐range order. This means that analysis of the EXAFS does not need extended long‐range order and in fact, even a single shell, e.g., the hydration sphere of solvated ions, can be investigated.

The analysis of the Mn‐L edges (and other first‐row transition metal L edges) can be performed similarly with some important differences. The involved transitions cannot be treated as a one‐electron process as the wave functions of the core and valence states overlap.^[89]^ Therefore, the Mn‐L edge corresponds to the system density of states (as opposed to the electron density of states measured at the Mn‐K edge) and the overlap gives rise to multiplet effects for the bound final states, similarly to the pre‐edges of K edges. Yet, the multiplet peaks are much larger in Mn‐L edge spectra and the main edge is barely visible. This can be seen clearly, e.g., in the Fe‐L edge spectra of ferricyanide,^[90]^ whose features are well understood.^[91]^ Due to the dependence on the multiplet structure and charge transfer effects, the Mn‐L edges depend strongly on the coordination symmetry and also on the number of d electrons, i.e., the oxidation state, similarly to the pre‐edges of K edges. The oxidation state is often determined empirically by calibration of the multiplet peak areas or peak to known reference materials. Alternatively, PCA and LCA are also used for L edge analysis.^[92]^ The EXAFS is also less visible at the L edges due to the small spin–orbit coupling^[93]^ and its analysis is severely restricted by the other L edges of the same element, e.g., the energy difference between the Mn‐L_3_ and Mn‐L_2_ edge (of Mn metal) is only 11.2 eV. It is rarely recorded and requires special efforts in the data refinement such as joint treatment of multiple edges.^[94]^

In summary, the average metal oxidation state is obtained from analysis of the XANES, while structural insights of the most frequent motifs are obtained from analysis of the EXAFS, even for samples without long‐range order. Since the attenuation length of the incoming (and outgoing) photons is much lower in water as compared to Mn oxides (Figure S1), it is well suited for in situ measurements in thin layers of aqueous solutions using the transmission or fluorescence modes. Insight into the electronic and geometric structure in a single experiment makes XAS an ideal complementary method to resolve ambiguities in electrochemical experiments.

## 4 When and How Do Electrocatalysts Change?

What can alter a freshly synthesized sample? In the worst case, simply exposure to our atmosphere as well as any subsequent sample preparation step and experiment performed. We have summarized the possible changes and when they may occur in Table 3. The processes leading to changes and examples are discussed in the following subsections. They are presented according to the moment when these are investigated with respect to the catalytic process: before (pre‐catalysis investigations), during (in situ and operando investigations) and after (post‐mortem investigations) catalysis.

**Table 3:** Possible changes of as‐prepared samples before, during and after electrocatalysis.

When does the electrocatalyst change?	Nature of change
During electrode preparation and storage	Chemical reaction
Immersion of electrodes in electrolyte	Chemical reaction
In electrolyte under polarization^[a]^	Electrochemical reaction
During/after catalysis^[b]^	Chemical & electrochemical reaction

[a] Not exposed to catalytic potentials. [b] Exposed to catalytic potentials.

### 4.1 Pre‐Catalysis Investigations

**Figure 5:**
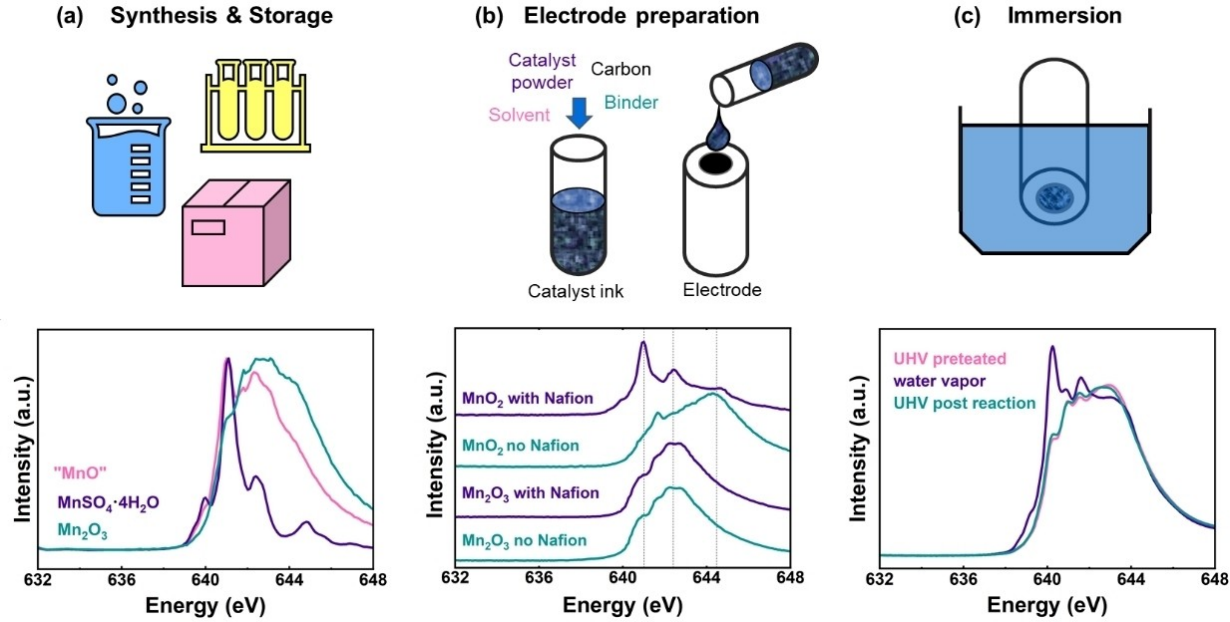
Possible changes that electrocatalysts undergo due to a) storage, b) electrode preparation and c) immersion in an electrolyte. a) Mn‐L_3_ edge of nominally MnO that shows unexpected oxidation. Mn‐L_3_ spectra of MnSO_4_⋅4 H_2_O and Mn_2_O_3_ are shown for comparison. They were recorded at the SGM beamline at Canadian Lightsource (CLS) in electron yield mode. b) Mn‐L_3_ edges of electrode films containing Mn oxide with and without Nafion showing reduction of Mn^4+^ in the presence of Nafion. c) Mn‐L_3_ edge of a Mn‐containing perovskite oxide showing reduction in water vapor. Panel (a) was modified from ref. [95] with permission from the ACS, panel (b) is adapted under CC BY 4.0 licence from ref. [95] and panel (c) is adapted under CC BY 3.0 license from ref. [96] Dataset in ref. [72].

The surfaces of many transition metal oxides, including common Mn oxides, react with their environment after synthesis, which could be due to exposure to oxygen in our atmosphere, to solvents and/or chemicals during electrode preparation, e.g., when preparing an electrocatalyst ink from powders, or to the electrolyte when the transition metal oxides are immersed and thus mounted in the electrochemical cell (Figure 5). As these changes are most pronounced near the electrocatalyst surface, we focus on Mn‐L edge XAS where the near surface region contributes more to the signal as compared to Mn‐K edges.

An example of a surface reaction due to storage is given by rocksalt MnO, where Mn is expected to be in oxidation state 2+. A typical spectrum of Mn^2+^ has a main peak at 641.1 eV with two minor peaks at 640.0 eV and 642.4 eV as found in the Mn‐L_3_ edge spectrum of MnSO_4_⋅4 H_2_O. Note that while the absolute energy values depend on the energy calibration, which is not harmonized in the field, the relative energy values and the fingerprint are still a valid diagnostic. Yet, the Mn‐L_3_ spectrum of “MnO” looks drastically different to that of MnSO_4_⋅4 H_2_O, and is composed of a superposition of the peaks of Mn^3+^ oxides (such as Mn_2_O_3_) and Mn^2+^ oxides (such as MnSO_4_⋅4 H_2_O), as shown in Figure 5a. The presence of the features from both oxidation states suggests the partial oxidation of the near surface. In mild cases of oxidation, the Mn‐K edge looks as expected for Mn^2+^ since oxidation only takes place at the electrocatalyst surface. However, in severe cases, oxidation may also lead to changes in the Mn‐K edge. An example for this are metallic Mn foils that not only show the spectrum of Mn^2+^ in Mn‐L edge XANES, but also display an additional shoulder in the Mn‐K edge XANES. Thus, we recommend to check physicochemical properties of the samples periodically, particularly for long storage times, and ideally before an electrochemical measurement.

Ink‐casting is a popular method to prepare electrodes for electrocatalytic investigations. The catalyst ink is typically prepared by mixing the oxide particles with a suitable dispersion medium such as an alcohol–water mixture or tetrahydrofuran (THF). Usually, further additives are added, namely high surface area carbon to enhance the electrode conductivity and a binder, typically an ionomer, to ensure mechanical stability of the electrode film. However, when preparing catalyst inks, some considerations are needed but often overlooked. Some transition metal oxides react with the dispersing solvent,^[97]^ in which case a different solvent should be used. Carbon may accelerate the amorphization of some oxide surfaces.^[98]^ Additionally, carbon may oxidize electrochemically to CO_2_ at similar potentials as the OER and can act as a co‐catalyst,[^99^, ^100^] e.g., during oxygen conversion, so that carbon may affect the product currents in addition to inducing changes of the electrocatalyst material.^[101]^

Our example for Mn oxides is the reaction with the common ionomer binder Nafion (Figure 5b).^[95]^ Mn^4+^O_2_ reacted chemically with Nafion to form Mn^2+^, while (Mn^3+^)_2_O_3_ was unaffected by the addition of Nafion. A similar trend was seen for other Mn^4+^‐containing oxides. We speculated that Mn^4+^ interacts with the electron donor groups in the binder.^[95]^ It is currently unclear whether other highly oxidized transition metals may also undergo redox reactions with Nafion or whether other ionomers react similarly. Consequently, we recommend to check for physicochemical changes after catalyst ink preparation.

The as‐prepared electrodes may further react prior to the desired electrochemical experiment due to immersion in the electrolyte. Many transition metal oxides are prone to react chemically with water (being beneficial to start the catalytic cycle of the OER). For example, Pr_0.2_Ca_0.8_Mn^3.8+^O_3_ reacted with water vapor to produce a Mn^2+^ species (Figure 5c). Other transition metal oxides, in particular those containing Ni, are known to incorporate Fe from alkaline electrolytes, which strongly modifies their electronic and thus catalytic properties.[^102^, ^103^, ^104^, ^105^] Also, in the absence of Fe, exposure to hydroxide solutions may change the surface and even the bulk phase of transition metal oxides after sufficiently long exposure.^[106]^ Finally, elements may dissolve from the electrocatalyst into the electrolyte changing the composition and thereby other properties, such as redox potentials or the catalytic reaction's overpotential.[^9^, ^107^, ^108^] Dosaev et al.^[109]^ recently studied Mn‐based spinels as synthesized, in the ink suspension and after soaking in hydroxide electrolyte, which oxidized Mn_3_O_4_ but not MgMn_2_O_4_. We recommend similar control experiments of the electrode soaked for an extended time (at least for the same duration of the intended experiment duration, but ideally much longer) to elucidate possible changes prior to electrochemical experiments.

### 4.2 Post‐Mortem Investigations

To investigate how the state of an electrocatalyst changes due to applied potential, i.e., due to electrochemical reactions, it is common to resort to post‐mortem experiments as some electrochemical changes can be resolved in this kind of investigations, namely those that result in the formation of stable phases. XAS is particularly useful for this purpose, as it allows to elucidate phase changes even in cases where amorphization takes place, which is not the case for techniques in which crystallinity is a prerequisite, e.g., diffraction‐based techniques.

**Figure 6:**
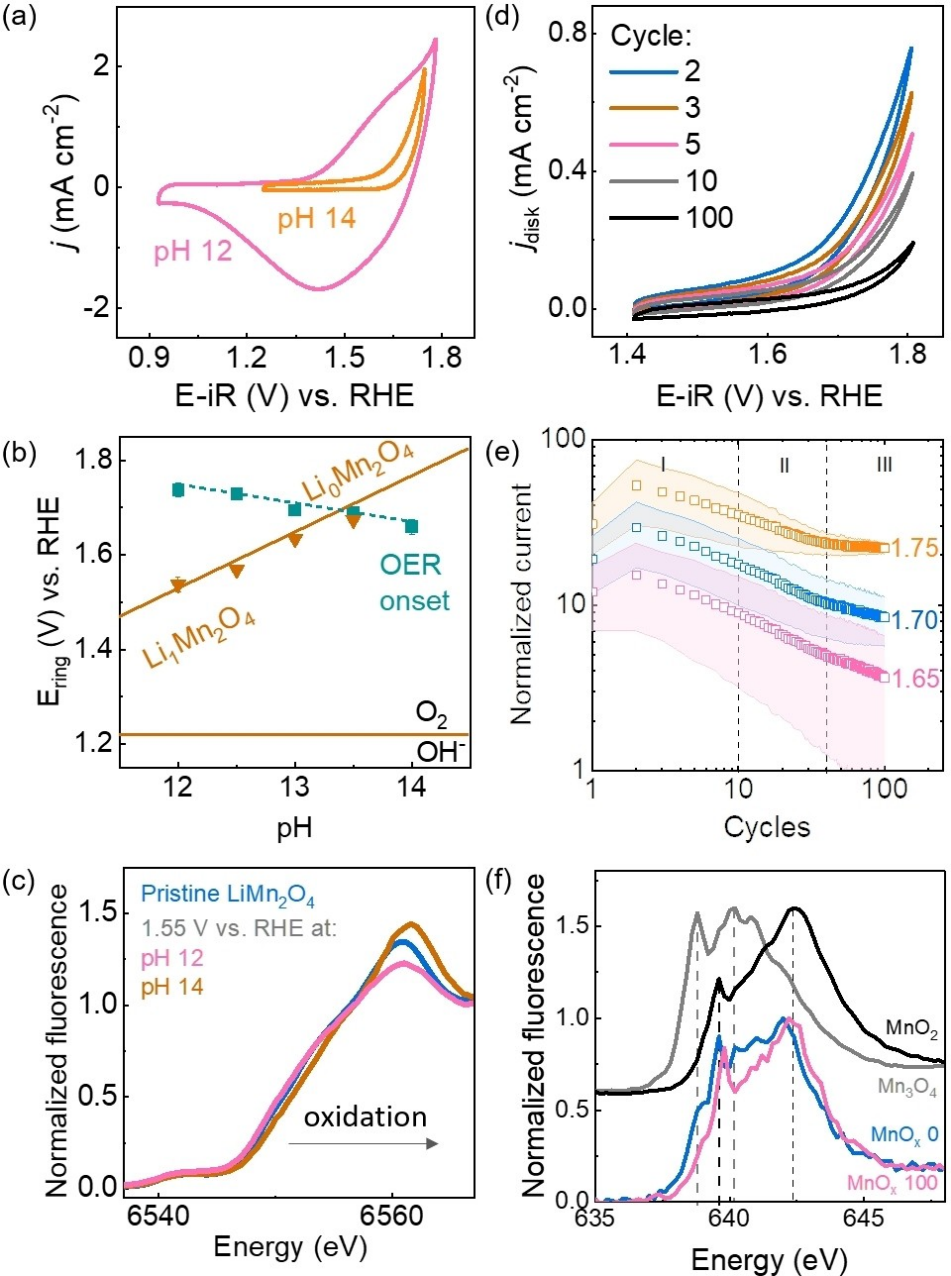
Post‐mortem investigations of electrocatalyst changes by XAS. a) CV (5^th^ cycle) of Li_*x*_Mn_2_O_4_ in 10 mM LiOH (pH≈12) and 1 M LiOH (pH≈14) showing large differences in redox currents. b) Delithiation potential (orange line) and OER onset (green line) extracted from the CVs. c) Mn‐K edge pristine and post‐mortem after conducting the OER at pH 12 and pH 14 in a chronoamperometric experiment. d) CV of an electrodeposited MnO_*x*_ film during 100 cycles in NaOH 0.1 M. e) Trend of normalized current in the CVs at 3 selected potentials of an electrodeposited Mn oxide film, and f) comparison between the film before voltammetric cycling (blue) and after recording 100 cycles (pink). Panels (a)–(c) have been modified from ref. [110] under CC BY 4.0 license and panels (d)–(f) have been modified from ref. [50] under CC BY 4.0 license.

The composition may change electrochemically without significantly affecting the geometric structure. This is a typical scenario for charging or discharging the bulk of a battery material but it is less discussed in the field of electrocatalysis, yet, several typical battery materials such as LiCoO_2_, LiMPO_4_ (M=Mn, Fe, Co) and LiMn_2_O_4_ have been investigated as electrocatalysts for the OER.[^47^, ^110^, ^111^, ^112^, ^113^, ^114^] In our example, we studied the OER on Li_*x*_Mn_2_O_4_ (Figure 6a,b) using a rotating‐ring disk electrode setup where the ring electrode qualitatively detected the oxygen produced at the disk electrode.^[110]^ The redox potential for delithiation, i.e., Li_1_Mn^3.5+^_2_O_4_→Li_0_Mn^4+^_2_O_4_, can be calculated taking into consideration the electrolyte composition (i.e., pH and Li concentration). Large redox peaks were observed in the CV at pH 12 while they were absent at pH 14 (Figure 6a),^[110]^ which suggests oxidation before the onset of the OER only at pH 12. The onset of the OER taking place at the disk electrode (defined therein as the potential at which 5 μA were reached at the ring electrode) shifted to more anodic potentials upon decreasing the pH, translating into lower OER activity, with higher potential for delithiation (Figure 6b). Baumung et al.^[110]^ prepared samples for post‐mortem XANES analysis which showed the expected oxidation (of the bulk) as an edge shift to higher photon energy for high delithiation overpotential (labeled pH 12 in Figure 6c). As a control experiment, a sample where the delithiation is not expected to occur also did not show an edge shift (labeled pH 14 in Figure 6c). We conclude that the results agree with other literature reports[^115^, ^116^, ^117^] where an oxidation of the metal site approaching Mn^4+^ reduces the activity for the OER.

Conditioning by potential cycling is a common procedure to activate catalysts prior to electrocatalytic studies.[^9^, ^118^, ^119^, ^120^, ^121^] The electronic and geometric structure of the activated electrocatalyst often differs from the as‐prepared electrocatalyst. As an example, we investigated the change in normalized current with voltammetric cycling for electrodeposited Mn oxide (Figure 6d,e).^[50]^ A current decrease was observed in the CV with cycling at all investigated voltages without change in the observed features (Figure 6d). Subsequently, the total current was divided by the capacitive current to normalize for differences in surface area so that any observed changes are most likely due to chemical changes. The normalized current was then plotted as a function of cycling at selected potentials (Figure 6e). At 1.75 V vs. RHE (a potential at which the OER takes place), a constant current is reached after about 40 cycles, while at less anodic potentials it decreases continuously upon cycling. While no significant changes were found in the Mn‐K edge, the Mn‐L_3_ edge (Figure 6f) showed clear spectral changes that suggested oxidation of the near‐surface toward Mn^4+^ by comparison of the activated electrocatalyst (100 cycles) with a spectrum of Mn^4+^O_2_. We concluded that the activation procedure led to oxidation of the electrocatalysts, with the changes being restricted to near the surface where catalysis occurs.^[50]^ Similar post‐mortem studies after electrocatalysis are commonly performed to elucidate the nature of the activated catalysts.^[9]^

### 4.3 In Situ Investigations

In situ or even operando experiments are necessary to understand both reversible and irreversible changes of the electrocatalyst as well as reactive transient states (if the time resolution is appropriate). Any combined EC and XAS experiment on transition metal oxides for studying the OER at room temperature is an in situ spectroscopic experiment as it needs to be conducted in an electrochemical cell including electrolyte and electrodes. In our definition, the reaction (e.g., the OER) must be taking place during a spectroscopic operando experiment^[122]^ and this needs to be proven by a qualitative or quantitative measurement of the product. For an electrocatalytic study, this could be the measured current if the Faradaic efficiency is known, or ideally, a simultaneous direct or online detection of the catalytic product. Thus, we use a stricter but clear definition of an electrocatalytic operando experiment as compared to the often found and somewhat ambiguous “electrocatalyst under reaction conditions”.

**Figure 7:**
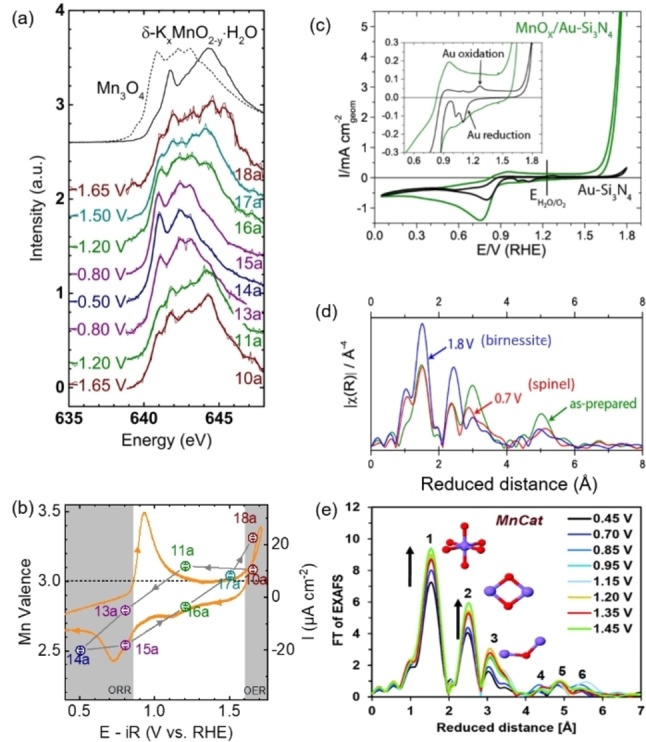
In situ investigations of electrocatalyst changes with applied potential. a) Mn‐L_3_ edge spectra of electrodeposited MnOx in 0.1 M KOH cycled from 1.65 V to 0.80 V and back to 1.65 V vs. RHE. b) Overlap between the CV of MnO_*x*_ in 0.1 M KOH and the valence changes during from subsequent chronoamperometry experiments at selected potentials showing hysteresis. c) Cyclic voltammetry of an electrodeposited MnO_*x*_ film in 0.1 M KOH for studies at the Mn‐K edge. d) EXAFS spectra collected on these MnO_*x*_ films as prepared, and after exposure to an OER potential (features of birnessite‐type) and an ORR potential (features of spinel‐type). e) Mn‐K edge EXAFS of electrodeposited MnO_*x*_ (MnCat) in 0.1 M KPi (pH 7) for potentials between 0.45 V to 1.45 V vs. NHE (0.04 to 1.04 V vs. RHE). Panels (a) and (b) are reproduced from ref. [27] with permission from the ACS. Further permission related to these figures should be directed to the ACS. Panels (c) and (d) were reprinted with permission from ref. [124], copyright 2013 American Chemical Society. Panel (e) was reproduced from ref. [117] with permission from the Royal Society of Chemistry.

As expected from the *E*–pH diagrams (Figure 3), transition metal oxides such as manganese oxide are oxidized and may undergo changes in their structural features under reaction conditions. Our example follows the changes occurring in the Mn‐L_3_ edge XAS of electrodeposited MnO_*x*_ from 1.65 V vs. RHE, a potential where the OER takes place, to 0.80 V vs. RHE, a potential where the ORR takes place, and back to 1.65 V vs. RHE (Figure 7a). The example also illustrates finer voltage resolution as compared to post‐mortem studies, which mainly compare before and after some known process such as an electrocatalytic reaction or a redox process. The spectra in Figure 7a clearly displayed reversible changes between that of the δ‐K_*x*_MnO_2_ reference (birnessite) recorded at 1.5 and 1.65 V vs. RHE and that of Mn_3_O_4_ (spinel) recorded at 0.5 and 0.8 V vs. RHE. Note that the spectra are susceptible to bubble formation during the OER at high potentials (e.g., spectrum 18a). The spectra collected at 1.2 V vs. RHE look similar to either that of birnessite or spinel depending on which was measured before. This indicates hysteresis due to slow kinetics of the phase changes and highlights the importance of the history of the electrode. The hysteresis can also be seen clearly when the oxidation state is calculated by calibration of the centroid under the Mn‐L_3_ edge with respect to reference materials (Figure 7b). Hysteresis of the Mn oxidation state with applied potential is known in the electrochemical capacitor community^[123]^ but rarely discussed in the field of electrocatalysis despite the popularity of cyclic voltammetry for electrocatalyst investigations. A CV recorded before the chronoamperometric in situ XAS measurements showed a clear redox peak with a midpoint potential at 0.83 V vs. RHE. At higher voltages, the Mn valence is about 3+, while it is below 3+ at lower voltages, so that the redox peak can be assigned to the Mn^2+/3+^ redox couple (Figure 7b). The analysis in a wider voltage range further shows that Mn reduction precedes the ORR, while Mn oxidation precedes the OER. Thus, both active states differ markedly from the as‐prepared oxide.

Changes in the oxidation state can also be resolved by Mn‐K edge XAS for samples with high surface to volume ratio such as porous films^[125]^ or nanoparticles.^[126]^ In situ XAS is especially powerful when metastable phases need to be studied such as the nucleation of MnO_2_.^[127]^ Previously unknown metastable phases have also been identified such as shown for α‐CoO_2_H_1.5_⋅0.5 H_2_O using PCA in a recent report, which could not be prepared for post‐mortem investigations as they only exist in situ.^[128]^

When transition metal oxides undergo redox reactions, their interatomic distances change accordingly. As discussed earlier, a change in edge position, and thus oxidation state, is expected to be proportional to 1/*R* ^2^ so that higher oxidation leads to shorter M−O bonds. This must also lead to the shortening of other interatomic distances, e.g., M−M, and/or other changes to the structure of solids, e.g., the connectivity of the coordination polyhedra. EXAFS analysis is ideally suited to resolve these changes.

Figure 7c shows the CV of electrodeposited MnO_*x*_ in 0.1 M KOH. It looks similar to the CV of the other electrodeposited Mn oxide in Figure 7b and also has the previously identified redox peak (inset). Analysis of the EXAFS at the Mn‐K edge shows the typical fingerprints of a birnessite‐type layered structure at OER potentials having a single peak at about 2.5 Å reduced distance and that of a spinel structure at ORR potentials having a double peak at similar reduced distance. Thus, the data show clearly that the Mn redox is accompanied by the structural change expected from the *E*–pH diagram (Figure 3), namely formation of a spinel phase at low potential.

In our next example of an electrodeposited Mn oxide,^[117]^ the phase remains spinel‐like but the coordination number of Mn−O in the first coordination shell changed as well as the number of Mn–Mn interactions (Figure 7e), which was interpreted as changes in the number of μ‐oxo bridges in the oxide, i.e., a change in polyhedron connectivity. Similar changes were also reported for electrodeposited Co oxide.^[129]^

In summary, operando and in situ XAS is mainly performed to elucidate the expected electronic and structural changes with applied potential. Yet, the unexpected changes such as hysteresis in cyclic experiments or metastable intermediate phases show the true potential of combining XAS and electrochemical experiments.

## 5 Summary and Perspective

In this Minireview, we reviewed what XAS can tell us about the active state of Mn oxides during the OER with references to other first‐row transition metal oxides. In the introduction, we highlighted that the macroscopic insight from electrocatalysis can be combined with the atomistic insight from XAS, such as the local coordination environment or polyhedral connectivity. The formation of the electrocatalytic product creates a flow of electrons, i.e., an electric current. XAS measures the electrons in the outer shell of atoms, i.e., the oxidation state of the metal, so that the two can be correlated. In order to put the desired correlations on a sound scientific basis, we briefly introduced the fundamentals of electrochemistry where we discussed the key processes of redox changes, double layer charging and electrocatalysis, all of which may contribute to the measured currents. *E*–pH diagrams, also called Pourbaix diagrams, were introduced, highlighting their relevance for in situ XAS experiments as they can provide some guidance about what changes to expect for a given combination of pH and applied potential. We continued to discuss the X‐ray absorption processes that create the analyzed features in XAS, namely the edge shift and EXAFS analysis of interatomic distances as well as number of scattering atoms. Based on this foundation, we discussed how XAS was used to identify electrocatalyst changes of the oxidation state due to storage, electrode preparation and immersion in an electrolyte. Irreversible changes of the oxidation state and local coordination environment during electrocatalyst activation can be detected by post‐mortem XAS, while in situ XAS allows finer voltage (or time) resolution to follow expected changes. Moreover, in situ XAS enables the study of metastable phases, as well as the investigation of reversible and reactive processes. In the following paragraphs, we will give a perspective on the operando XAS studies needed to close current knowledge gaps.

The relevant time scales of solid state and electrocatalytic processes differ drastically and both are not matched by most current XAS studies. Current conventional in situ XAS studies are performed with durations of a few minutes to an hour per spectrum, which is appropriate to study the faster changes in *E*–pH diagrams. While scan durations of the order of minutes are sufficient to study slow processes such as corrosion and bulk phase changes, the expected changes may occur on time scales longer than typical synchrotron experiments of one to two weeks, which is not compatible with usual synchrotron operation where users apply for an allotment of one or few measurement weeks. A possible solution might be implementing alternative operation modes where one applies for an allotment of a few hours each week over a long duration. Another solution could be the use of lab‐based XAS systems, albeit at the cost of lower X‐ray intensity. It will be crucial for the optimization of electrocatalytic materials with relevance for practical applications to understand the origins of degradation that often occur on time scales much longer than those currently investigated in most academic reports.

Typical current XAS scans are also too slow to resolve changes with relevance to the catalytic mechanism such as Mn redox changes of the order of tens to hundreds of milliseconds for electrodeposited Mn oxide.^[117]^ Quick EXAFS (QEXAFS) performed with a special monochromator reduces the scan time for a spectrum to seconds or even milliseconds.[^130^, ^131^] However, such scan time is too fast for the acquisition time of most fluorescence detectors. Thus, these experiments are performed in transmission mode, which is less common for electrocatalytic studies and technically challenging at metal L edges due to the absorption of water.^[132]^ Another approach to faster XAS is implemented using conventional monochromators fixed to a well‐selected excitation energy and measuring the change in intensity using appropriate detectors including fluorescence detectors. The method has been recently reviewed by Tesch and Simonov^[133]^ in the context of electrocatalytic reactions. It is commonly used to track the X‐ray signal during cyclic voltammetry but can also be used to record transients during potentiostatic measurements.^[134]^ These faster XAS measurements will be crucial to separate changes of the oxidation state due to non‐catalytic processes from those occurring during catalysis, and for precise identification of the redox states involved in the electrocatalysis of the OER and of other reactions.

**Figure 8:**
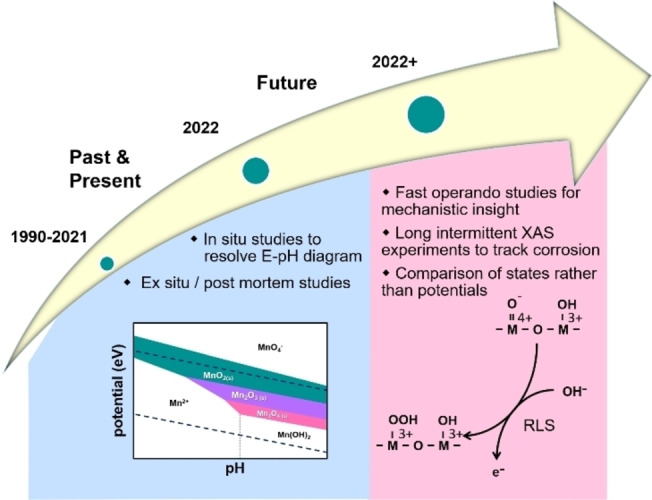
Possible evolution of combined electrochemical and XAS investigations. The *E*‐pH diagram was produced using materialsproject.org;^[53]^ under CC BY 4.0 license.

The final point that can advance future combined EC and XAS experiments is the study of states rather than applied potentials. Relevant states are the redox changes of transition metals such as Mn. They may be identified by electrochemical features in cyclic voltammograms (Figure 2a) but especially Mn oxides have redox transitions that do not result in obvious voltammetric features.^[62]^ These redox transitions can be resolved using spectroscopic methods, e.g., UV/Vis spectroscopy[^135^, ^136^] and XAS,[^27^, ^92^, ^124^, ^137^] which have signals proportional to the oxidation state (a charge, *Q*). The time derivative of the signals (d*Q*/d*t*) is thus the redox current.^[129]^ Alternatively, the redox couples can be identified by fitting the oxidation state from XAS to (modified) Nernst equations.^[129]^ The nature of the redox couples directly relates to an atomistic understanding of the key steps of the catalytic mechanism, e.g., of the rate‐limiting step as shown in Figure 8, and their midpoint potential is crucial to understand charge transfer from/to semiconductors, e.g., in the framework of the Marcus–Gerischer theory.[^138^, ^139^, ^140^] Identification of the relevant redox couples is thus a natural choice for mechanistic discussions and has led to the recent insight that the OER is a first‐order reaction with respect to the Mn density of states, while the ORR is of second order.^[141]^ Moreover, the Mn^3+/4+^ redox couple is essential for the evolution of oxygen in natural photosynthesis as well as for electrodeposited Mn oxides^[142]^ because Mn^4+^ together with Mn^3+^ has been proposed as a necessity for the OER and because its midpoint potential is similar to that of the OER. The M^3+/4+^ redox couple is likewise important for the OER on other transition metal oxides,^[142]^ where the detection of M^4+^ has been correlated with oxygen evolution for a Co oxide.^[129]^ Moreover, the midpoint potential of the Mn^3+/4+^ redox couple was also used to rationalize electrocatalytic trends of the ORR.^[143]^ We expect that combined operando EC–XAS studies will continue to unravel important mechanistic details of the OER and of other reactions as well as provide crucial physical insight to build improved models for the knowledge‐guided design of electrocatalysts.

## Conflict of interest

The authors declare no conflict of interest.

6

## Biographical Information

*Marcel Risch leads the Young Investigator Group NOME at Helmholtz‐Zentrum Berlin cofunded by the ERC Starting Grant ME4OER. He earned his PhD from Free University Berlin and performed interdisciplinary postdoctoral work at MIT. Marcel is enthusiastic about the knowledge‐guided design of electrocatalysts for oxygen and nitrogen electrocatalysis for sustainable fuels such as green hydrogen harnessing insights from innovative operando experiments*.

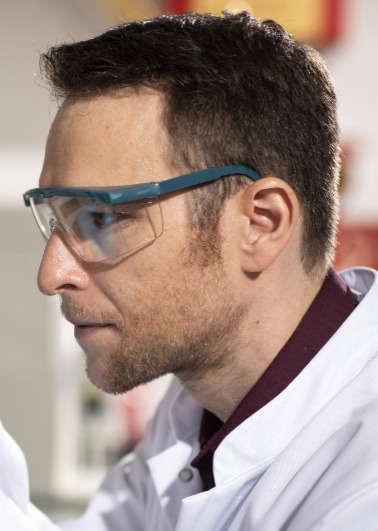

## Biographical Information

*Dulce M. Morales is currently deputy head of the Young Investigator Group NOME led by Dr. Marcel Risch at Helmholtz‐Zentrum Berlin. She has a background in Industrial Chemical Engineering from Instituto Politécnico Nacional (Mexico), and earned her doctoral degree in Chemistry in 2019 mentored by Prof. Wolfgang Schuhmann at Ruhr‐Universität Bochum. Her research focuses on the development of multi‐metallic composites as electrocatalysts for sustainable energy conversion and storage applications, and presently aims for their study under industrial‐relevant conditions*.

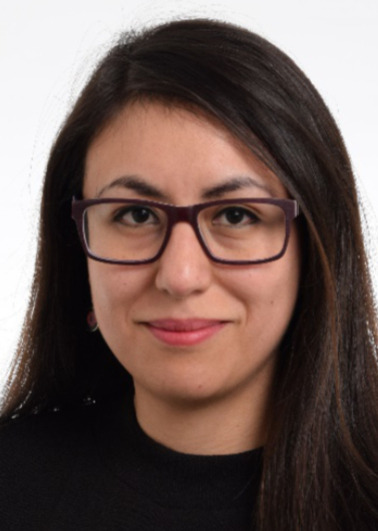

## Biographical Information

*Javier Villalobos is currently a Ph.D. student in the Young Investigator Group “Oxygen Evolution Mechanism Engineering” led by Dr. Marcel Risch at Helmholtz‐Zentrum Berlin. He has a background in Chemistry from the University of Costa Rica, where he obtained his master's and bachelor's degrees. His research focuses on studying the stability and activation of Co‐ and Mn‐based electrocatalysts for oxygen evolution reaction by X‐ray spectroscopy, including the development of operando experiments*.

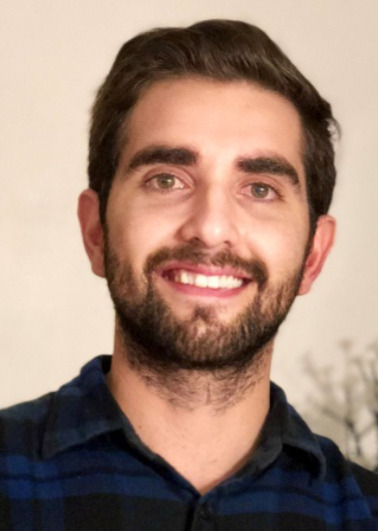

## Biographical Information

*Denis Antipin graduated from Lomonosov Moscow State University with a master's degree in Materials Science. He is currently a Ph.D. student in the Young Investigator Group “Oxygen Evolution Mechanism Engineering” led by Dr. Marcel Risch at Helmholtz‐Zentrum Berlin. His current research interests include simulations and experimental investigation of the mechanistic parameters of the oxygen evolution reaction*.

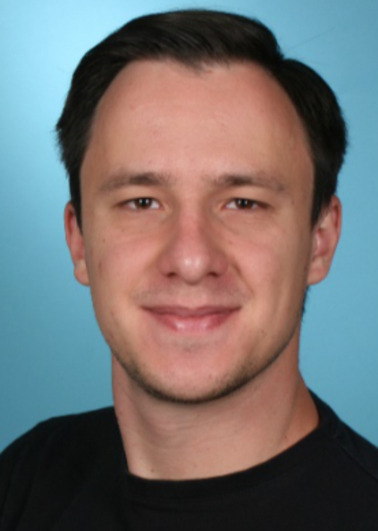

## Supporting information

As a service to our authors and readers, this journal provides supporting information supplied by the authors. Such materials are peer reviewed and may be re‐organized for online delivery, but are not copy‐edited or typeset. Technical support issues arising from supporting information (other than missing files) should be addressed to the authors.

Supporting Information

## Data Availability

The reference data that amend the published works of this study are openly available in Figshare at https://doi.org/10.6084/m9.figshare.20393064, reference number 20393064. For other data refer to the original publication.
