# Supplementary material for: What X‐Ray Absorption Spectroscopy Can Tell Us About the Active State of Earth‐Abundant Electrocatalysts for the Oxygen Evolution Reaction
Source: Angew Chem Int Ed Engl. 2022 Nov 15;61(50):e202211949. doi: 10.1002/anie.202211949 (PMC10100097; doi:10.1002/anie.202211949)
Supplement: Supplementary file 1 — Supporting Information [file ANIE-61-0-s001.pdf]

Angewandte Chemie
Eine Zeitschrift der Gesellschaft Deutscher Chemiker

Supporting Information

What X-Ray Absorption Spectroscopy Can Tell Us About the Active State of Earth-Abundant Electrocatalysts for the Oxygen Evolution Reaction

M. Risch, D. M. Morales, J. Villalobos, D. Antipin*

Supporting Information

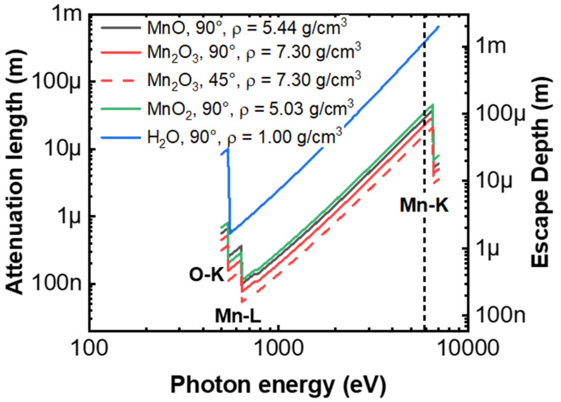

Figure S1. Calculated attenuation lengths of the Mn oxides MnO, Mn_2O_3 and MnO_2 as well as water. The calculation was performed using the Henke Tables[1] and densities were estimated. 90° incidence is typical for transmission measurements while 45° incidence is typical for fluorescence measurements. The dashed line indicates the Mn-Ka line, which is used to calculate the attenuation length in fluorescence experiments (at the Mn-K edge). We estimated the escape depth as trice the attenuation length (i.e., 99.7 % attenuation). Dataset in ref. [2]

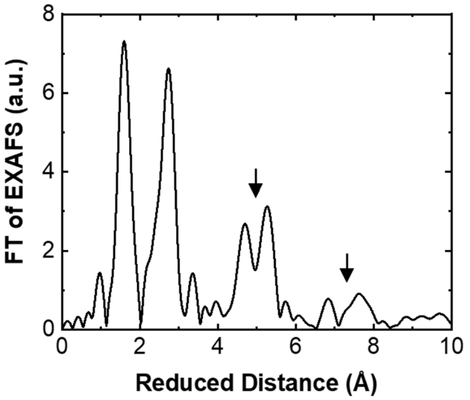

Figure S2. Fourier Transform (FT) of the EXAFS of commercial $LiMnO_2$ (Sigma Aldrich) measured in transmission mode at the ALBA synchrotron. For the measurement, a pellet of $LiMnO_2$ diluted with boron nitride (5 wt%) was pressed and encapsulated in Kapton foil. The arrows indicate FT peaks due to constructive interference in the ordered crystal structure. Dataset in ref. [2]

References

1 B. L. Henke, E. M. Gullikson and J. C. Davis, *At. Data Nucl. Data Tables*, 1993, **54**, 181–342.

2 M. Risch, D. M. Morales, J. Villalobos and D. Antipin, 2022, DOI: 10.6084/m9.figshare.20393064.

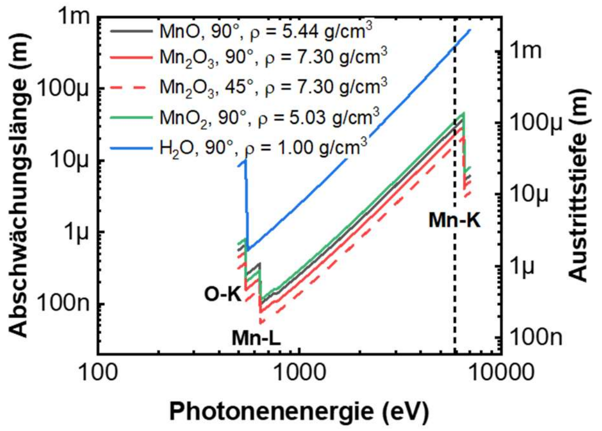

Abbildung S1. Berechnete Abschwächungslängen der Mn-Oxide MnO, Mn_2O_3 und MnO_2 sowie von Wasser. Die Berechnung erfolgte anhand der Henke-Tabellen,[1] und die Dichten wurden geschätzt. Ein 90°-Einfall ist typisch für Transmissionsmessungen, während ein 45°-Einfall typisch für Fluoreszenzmessungen ist. Die gestrichelte Linie zeigt die Mn-Ka-Linie an, die zur Berechnung der Abschwächungslänge bei Fluoreszenzexperimenten (an der Mn-K Kante) verwendet wird. Wir schätzten die Austrittstiefe auf das Dreifache der Abschwächungslänge (d. h. 99,7 % Abschwächung). Datensatz in Ref. 2

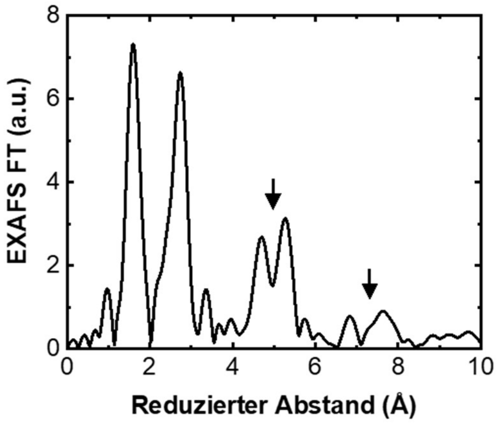

Abbildung S2. Fourier-Transformation (FT) der EXAFS von kommerziellem $LiMnO_2$ (Sigma Aldrich), gemessen im Transmissionsmodus am ALBA-Synchrotron am CLÆSS Strahlrohr. Für die Messung wurde ein mit Bornitrid (5 Gew.-%) verdünnter $LiMnO_2$-Pressling Hergestellt und in Kaptonfolie eingekapselt. Die Pfeile zeigen FT-Peaks an, die auf konstruktive Interferenzen in der geordneten Kristallstruktur zurückzuführen sind. Datensatz in Ref. 2

Literaturquellen

1 B. L. Henke, E. M. Gullikson and J. C. Davis, *At. Data Nucl. Data Tables*, 1993, **54**, 181–342.

2 M. Risch, D. M. Morales, J. Villalobos and D. Antipin, 2022, DOI: 10.6084/m9.figshare.20393064.
